# Parvalbumin interneuron deficiency in the prefrontal and motor cortices of spontaneously hypertensive rats: an attention-deficit hyperactivity disorder animal model insight

**DOI:** 10.3389/fpsyt.2024.1359237

**Published:** 2024-03-27

**Authors:** Ewelina Bogdańska-Chomczyk, Maciej Równiak, Andrew Chih-Wei Huang, Anna Kozłowska

**Affiliations:** ^1^Department of Human Physiology and Pathophysiology, School of Medicine, University of Warmia and Mazury, Olsztyn, Poland; ^2^Department of Animal Anatomy and Physiology, Faculty of Biology and Biotechnology, University of Warmia and Mazury, Olsztyn, Poland; ^3^Department of Psychology, Fo Guang University, Yilan County, Taiwan

**Keywords:** attention-deficit/hyperactivity disorder, cortex, parvalbumin, dopamine receptor, tyrosine hydroxylase, spontaneously hypertensive rat, GABAergic system, animal model

## Abstract

**Background:**

Attention deficit hyperactivity disorder (ADHD) is characterized by impairments in developmental–behavioral inhibition, resulting in impulsivity and hyperactivity. Recent research has underscored cortical inhibition deficiencies in ADHD via the gamma-aminobutyric acid (GABA)ergic system, which is crucial for maintaining excitatory–inhibitory balance in the brain. This study explored postnatal changes in parvalbumin (PV) immunoreactivity, indicating GABAergic interneuron types, in the prefrontal (PFC) and motor (MC) cortices of spontaneously hypertensive rats (SHRs), an ADHD animal model.

**Methods:**

Examining PV- positive (PV+) cells associated with dopamine D2 receptors (D2) and the impact of dopamine on GABA synthesis, we also investigated changes in the immunoreactivity of D2 and tyrosine hydroxylase (TH). Brain sections from 4- to 10-week-old SHRs and Wistar Kyoto rats (WKYs) were immunohistochemically analyzed, comparing PV+, D2+ cells, and TH+ fiber densities across age-matched SHRs and WKYs in specific PFC/MC regions.

**Results:**

The results revealed significantly reduced PV+ cell density in SHRs: prelimbic (~20% less), anterior cingulate (~15% less), primary (~15% less), and secondary motor (~17% less) cortices. PV+ deficits coincided with the upregulation of D2 in prepubertal SHRs and the downregulation of TH predominantly in pubertal/postpubertal SHRs.

**Conclusion:**

Reduced PV+ cells in various PFC regions could contribute to inattention/behavioral alterations in ADHD, while MC deficits could manifest as motor hyperactivity. D2 upregulation and TH deficits may impact GABA synthesis, exacerbating behavioral deficits in ADHD. These findings not only shed new light on ADHD pathophysiology but also pave the way for future research endeavors.

## 1 Introduction

Attention deficit hyperactivity disorder (ADHD) is a complex neurodevelopmental condition affecting the central nervous system characterized by persistent inattention, hyperactivity, and impulsivity throughout an individual’s life span (1, 2). It is believed to be a childhood disorder; but long-term follow-up studies revealed that symptoms often persist into adulthood (3, 4). The precise etiology and pathogenesis of ADHD remain elusive, with increasing evidence implicating stress, anxiety, and neuroinflammation in the emergence of this disorder (5, 6). For example, increased inflammation could lead to changes in the synthesis, release, or clearance of a number of neurotransmitters (7–9). The etiology of the disorder involves the dysregulation of various neurotransmitters, including dopamine (DA), norepinephrine (NA), and serotonin (5-HT).

ADHD is intricately linked to dysregulation in DA neurotransmission, a key factor influencing attention, reward, and motivation within neural circuits spanning the prefrontal cortex (PFC) and subcortical structures such as the basal ganglia (10–12). Dysfunctions in the DA receptors D1 and D2, anomalies in dopamine transporters, and perturbations in DA release and synthesis underscore the molecular intricacies contributing to ADHD symptoms (13). In addition, the interplay between the inhibitory gamma-aminobutyric acid (GABA)ergic system and dopaminergic signaling pathways is crucial for regulating the cognitive and motor functions in the brain, both of which are impaired in ADHD (14, 15). GABAergic neurons, utilizing GABA as their principal neurotransmitter, exert inhibitory control on neural circuits, preventing excessive excitability and contributing to the balance of neuronal activity. Dopaminergic signaling, driven by DA, modulates various physiological functions, including cognition and motor control (15). The intricate reciprocal modulation between the GABAergic inhibitory neurons and dopaminergic pathways occurs in cortical and subcortical regions, creating a dynamic regulatory network (16, 17).

Dysregulation in this interplay has been implicated in neurological and psychiatric disorders, including ADHD (18–20). There is a suggestion, supported by recent findings, that a deficit in behavioral inhibition may be at the core of ADHD (21–23). For instance, evidence indicates a reduction in the concentration of the principal inhibitory neurotransmitter GABA in certain studied cortical (22, 24) and subcortical (23) brain regions of children with ADHD. Reduced GABA content has also been reported in the hippocampus of the animal model of ADHD, spontaneously hypertensive rats (SHRs) (25). Studies in animal models are pivotal in translational research, offering insights into the biological and physiological processes pertinent to human health and the comprehension of numerous disorders (26). Moreover, recent studies in female ADHD patients have indicated that low GABA concentrations in the PFC were strongly associated with high inattention scores (24). In addition, diminished short-interval cortical inhibition (SICI), influenced by GABA-A agonists, is correlated with the severity of ADHD symptoms and motor skills (27). Although the cellular components involved in this phenomenon are still unclear, parvalbumin (PV)-expressing (PV+) cells appear to be quite good candidates as they use GABA (28) and have an abundant amount of GABA-A receptors (29).

The behavioral inhibition mediated by GABAergic interneurons in the cortex is modulated by DA, as evidenced by the influence of dopaminergic innervation on GABAergic neurons (30–34) and the abundant localizations of DA receptors in these cells (33). Moreover, current evidence shows that DA inhibits both spontaneous and evoked neural activity in the PFC, and there is good evidence that this inhibition is mediated by GABAergic neurons (34–36). Notably, these interneurons, which receive the heaviest dopaminergic innervation and possess the most abundant DA receptors, are PV+ cells that exert potent inhibitory actions on pyramidal cells (35). Given the capability of a single GABAergic interneuron to synapse on hundreds of pyramidal cells, the activation of a limited number of these interneurons by DA is sufficient to induce strong local cortical inhibition (37, 38). However, in ADHD, the downregulation of tyrosine hydroxylase (TH), a rate-limiting enzyme in DA synthesis, has been observed in patients and in animal models, potentially impacting GABA neurotransmission and exacerbating deficits in behavioral inhibition (39). GABAergic cells, including PV+ cells, express both D1 (activating) and D2 (deactivating) DA receptors. Dysregulation of these receptors could lead to severe alterations in GABA synthesis and contribute to abnormalities in behavioral inhibition in individuals with ADHD (33).

In light of these findings, our hypothesis posits that ADHD-affected individuals demonstrate a) diminished GABA activity attributable to deficits in PV+ neurons in cortical regions and b) perturbations in DA activity due to a diminished density of TH+ fibers and DA receptor (D2)+ cells. To test this hypothesis, the densities of interneurons expressing PV (GABAergic), cells endowed with D2, and fibers expressing TH were compared in the PFC and motor cortex (MC) of SHRs, considered to be a validated animal model of ADHD, and Wistar Kyoto rats (WKYs), which served as the control strain (40). The PFC was chosen for investigation due to reported abnormalities in patients with ADHD, including morphological and circuit irregularities and weaker activation during attention and behavior regulation (41, 42). Moreover, lesions of the PFC produce symptoms that are quite similar to those observed in patients with ADHD (12). The MC was selected because either ADHD patients or SHRs display increased motor activity (43). PV was proposed as a marker of GABAergic interneurons as, in rats, PV+ cells comprise numerous populations of GABAergic interneurons in the mammalian cerebral cortex (44–46). It was estimated that PV+ interneurons make up roughly 40% of the total GABAergic interneuron population in the rat cerebral cortex (47–49). Finally, recent studies have indicated that PV+ neurons function as a cohesive unit, orchestrating activity within the local PFC circuit during goal-driven attentional processing (50). D2 was chosen based on evidence indicating its abundant expression on cortical PV+ neurons (33), suggesting its potential role in mediating hyperactivity in ADHD (51). TH was considered due to its role as a rate-limiting enzyme in DA synthesis and its downregulation in the PFC of SHRs (39) and in patients with ADHD (41). The study employed rats aged 4–10 weeks to cover the growth stages from weaning to adulthood, aligning with the manifestation of ADHD-related abnormalities in children and in prepubertal SHRs (52). Investigating both hemispheres separately addressed the observed hemisphere-specific abnormalities (53, 54). Due to the evident male bias in patients with ADHD and in SHRs, the study focused its investigation on males of this strain (55, 56).

## 2 Materials and methods

### 2.1 Subjects

In order to test the previously mentioned hypotheses, male SHRs (ADHD group) and WKYs (control group) at 4, 5, 6, 7, 8, 9, and 10 weeks of age were used as subjects in the present investigation (*n* = 5 or 6 per group). Animals from both groups at 21 days of life were purchased from Charles River (Germany) and conveyed to the animal facilities at the Institute of Animal Reproduction and Food Research of the Polish Academy of Sciences (Olsztyn, Poland). In this study, the animals were housed in sanitized polypropylene cages in pairs or in threes to avoid social isolation stress. The rats were maintained in climate-controlled rooms (21 ± 1°C, 12–20 exchanges/h) with diurnal lighting (12/12-h light/dark cycle: lights on at 8:00 hours, lights off at 18:00 hours). All rats had free access to their diet (VRF1 diet; Charles River, Germany) and tap water. All animal housing and handling were conducted in strict accordance with the European Union Directive (2010/63/EU). The use of animals and all protocols were approved by the Local Ethical Commission of the University of Warmia and Mazury in Olsztyn (no. 43/2014). Furthermore, all attempts were made to reduce animal suffering and decrease the number of animals to the minimum needed to yield accurate data. The rat strains used in this study have been carefully selected. The SHRs from Charles River (Germany) were chosen as behavioral, genetic, and neurobiological studies have indicated that these rats are at present the most appropriate model of ADHD (40). WKYs are the classical control for rat ADHD models (especially SHRs). In addition, the same rat strains have been evaluated by us (57) using the various behavioral tests that confirmed the symptoms of ADHD in SHRs. We have previously reported that exposure of SHRs to an open-field arena results in an increase in motor activity and a drop in the anxiety behavior of these animals. Thus, these rat strains were chosen for investigation in the present study. Moreover, the time points of the rat’s life span were deliberately selected. The decision to select animals at 4 weeks for investigation was based on the fact that prepubertal SHRs exhibit ADHD abnormalities and symptoms (58) while being free of hypertension (59). On the other hand, postpubertal and mature SHRs no longer display symptoms of ADHD (58), but develop hypertension (59).

### 2.2 Tissue preparation

After the habituation phase, all SHRs and WKYs were randomly allocated into seven age groups in accordance with the study plan, with all animals from each group later given the same tissue processing. Briefly, the rats were acutely anesthetized with an intraperitoneal inoculation of Morbital (Biowet, Puławy, Poland; 2 ml/kg, 133.3 mg/ml of pentobarbital sodium salt and 26.7 mg/ml of pentobarbital). Following cessation of breathing, animals were immediately perfused transcardially with saline (0.9%) followed by 4% paraformaldehyde (PFA; pH 7.4 (1040051000; Merck, Darmstadt, Germany), which was dissolved in phosphate-buffered saline (PBS) (P5493; Sigma-Aldrich, Schnelldorf, Germany). After perfusion, the whole brain was conscientiously removed from the skull. In the next step, brains were post-fixed by immersion in 4% PFA for 24 h and then rinsed three times in 0.1 M phosphate buffer (pH 7.4, 4°C). Thereafter, all brains were cryoprotected in series (10%, 20%, and 30%) with sucrose (363-117720907; Alchem, Wrocław, Poland) in 1× PBS at 4°C until they sunk (3–5 days). Conclusively, the brains were frozen as blocks and were then coronally cut at a thickness of 10 μm using a cryostat (HM525; Zeiss, Jena, Germany). The tissue sections were placed on glass slides and stored at −80°C until further investigation.

### 2.3 Immunohistochemistry

Chosen brain sections comprising the PFC and MC from both strains (SHRs and WKYs) were stained using two standard immunohistochemical methods: immunoperoxidase reaction targeted a neuron-specific nuclear protein (NeuN) and immunofluorescence focused on PV, D2, and TH. Both staining methods were performed in a special humid and dark chamber (Immuno Slide Staining Trays, R64001-E; Pyramid Innovation Ltd., Polegate, UK) at room temperature.

#### 2.3.1 DAB method

To define the location and borders of the various PFC and MC regions in the brain, every 25th brain section was bound to diaminobenzidine (DAB) labeling (Dako Liquid DAB + Substrate Chromogen System, K3468, Glostrup, Denmark), which was accurate in more detail in our previous studies (60, 61). Concisely, the slides with the selected brain sections were covered overnight with a solution of primary antibody: NeuN (anti-NeuN antibody, clone A60, MAB377, 1:1000 dilution; Merck Millipore, Warsaw, Poland). Subsequently, the sections were covered for 1 h with a solution of secondary antibodies (ImmPRESS™ Universal Reagent anti-mouse/rabbit IgG peroxidase, MP-7500, 1:1 dilution; Vector Laboratories Inc., Burlingame, CA, USA). Then, all brain sections were rinsed in PBS and covered for 1 min with a DAB substrate and chromogen mixture. Finally, labeled brain sections were washed in tap water, dehydrated in a series of alcohols (POCH, Gliwice, Poland), cleaned in xylene, and covered in DPX (DPX Mountant for histology, 44581; Sigma-Aldrich, Schnelldorf, Germany).

#### 2.3.2 Immunofluorescence

For neurochemical analysis, slides containing the selected brain sections composed of the PFC and MC were treated for the standard single immunofluorescence staining described earlier by Kozłowska et al. (61) These brain sections were covered overnight with a solution of primary antibodies: PV (mouse, cat. no. 235, 1:4000 dilution; Swant, Burgdorf, Switzerland), D2 (rabbit, cat. no. AB5084P, 1:1000 dilution; EMD Millipore, Billerica, MA, USA), or TH (mouse, cat. no. MAB 318, 1:1000 dilution; EMD Millipore, Billerica, MA, USA). Subsequently, they were washed in PBS (3 × 15 min) and then covered for 1 h with a solution of secondary antibodies: Alexa 488 (cat. no. A-21202, 1:1000 dilution; Thermo Fisher Scientific, Waltham, MA, USA) or Alexa 568 (cat. no. A-11011, 1:1000 dilution; Thermo Fisher Scientific, Waltham, MA, USA). Finally, all brain sections were covered with a fluorescent mounting medium (cat. no. S3023; Agilent, Glostrup, Denmark). Furthermore, to evaluate the relationship between PV+ neurons and cells enriched in D2, additional sections of the subject were processed for double immunofluorescence staining as described earlier by Równiak et al. (29). In this case, the sections were covered with a mixture of primary antibodies consisting of mouse antisera against PV and rabbit antisera toward D2, the same antibodies used in the single immunofluorescence experiment.

### 2.4 Controls

NeuN, stained using immunoperoxidase techniques, is a sensitive and specific neuronal marker for neurons in the peripheral and central nervous systems (62). The specificity of a mouse antibody against PV (235) and rabbit antibody against D2 (AB5084P) has also been proven by various researchers in multiple previous studies (63–66). Moreover, these antibodies have been positively evaluated using Western blotting, immunohistochemistry in knockout mouse brain sections, and immunoprecipitation, justifying their specificity to their targets (63, 64, 66). The mouse antibody toward TH (MAB 318) is commonly used in brain studies on dopaminergic neurotransmission (67, 68). The specificity of secondary antibodies was examined by the omission of the primary antibody and its replacement by nonimmune sera or PBS. The absence of any response designated their specificity.

### 2.5 Cell and fiber counting

To validate the aforementioned hypotheses, a single immunofluorescence staining method was employed. This widely utilized technique in molecular and cell biology laboratories is a robust and straightforward approach for accurately localizing molecules within a diverse array of fixed cells or tissues. Quantification of the density of neurons and/or immunoreactive fibers for PV, D2, and TH in the selected regions of PFC and MC was carried out using an Olympus BX61 microscope provided with the cellSens Dimension image analysis software (Olympus, Tokyo, Japan). The following PFC regions were analyzed: prelimbic (PRL), anterior cingulate (CG1), lateral orbitofrontal (LO), and ventral orbitofrontal (VO). Within the MC, the primary (M1) and secondary (M2) motor cortices were studied. For each PFC/MC area in each animal for both SHRs and WKYs, PV+ and/or D2+ cells and TH-positive fibers were manually counted on 10 evenly distributed sections. To confirm the localization of specific PFC/MC regions on the sections, they were stained with mouse anti-NeuN (pan-neuronal marker). All measurements on the individual section were made at ×40 magnification using 220 µm × 170 µm areas as the test frames. Based on the cross-section size of the specific PFC/MC area, scores were calculated from either one such field located in the middle of the area (covering 100% of its cross-sectional area) or two to six bordering on non-overlapping fields. All scores determined inside the test frames in the individual PFC/MC area on the section were averaged. As such, the mean density value was mentioned only for the region of the test frame and was recalculated every time to present the density of the neurons in 1 mm^3^ of the brain tissue. To determine the mean density of the neurons in the entire single PFC/MC area in the rat, the means of the individual sections were averaged. Eventually, the density values from every PFC/MC area were averaged for every age range in both SHRs and WKYs and shown as the mean ± standard deviation (SD). It should be underlined that all calculations were made on coded slides prepared by the first author. To abstain from fluorescence fading, every test frame was digitally recorded before estimation. Digitalized test frames were then assessed by two independent researchers who had no knowledge of the parameters of the tissue under study (i.e., strain, age, and PFC/MC region, among others). The scores of these calculations presented high inter-rater reliability using Pearson’s correlation test (*r* = 0.79, *p* < 0.05). It should be acknowledged here that the data for PRL and CG1 in the 5- and 10-week-old WKYs and SHRs shown in the current study were from our previously published paper (61). They were included here only to complete the pattern of postnatal development on the graphs.

### 2.6 Statistical analysis

Mean differences between multiple groups were comprehensively assessed through one-way ANOVA using GraphPad Prism 6 software (GraphPad Software, La Jolla, CA, USA). Following this, Tukey’s *post-hoc* test was conducted. The ability of Tukey’s test to adjust for multiple comparisons enhanced the reliability and interpretability of our findings. In addition, the potential presence of non-normally distributed data or situations where the assumptions of ANOVA may not be fully met was recognized, leading to the incorporation of the Mann–Whitney *U* test. This nonparametric test allowed confirmation or exclusion of observed differences between pairs. A *p* < 0.05 was assumed to indicate that the difference is statistically significant.

### 2.7 Photomicrographic production

Low-magnification photomicrographs of immunoperoxidase-stained sections were obtained by digitizing these sections with ×5 magnification using a PathScan Enabler IV Histology Slide Scanner (Prague, Czech Republic). High-magnification photomicrographs of the immunofluorescence-stained sections were taken using a CC-12 digital camera (Soft Imaging System, Münster, Germany) on an Olympus BX61 microscope.

## 3 Results

**Figure 1:**
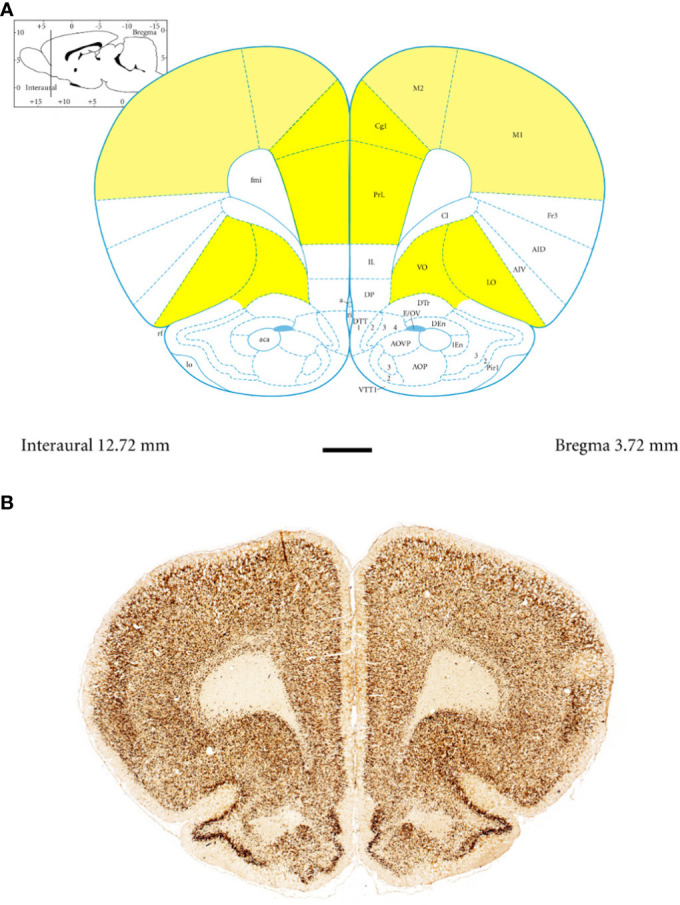
Topography and subdivisions of the prefrontal cortex (PFC, highlighted in *yellow*) and the motor cortex (MC, *light yellow*). **(A)** Schematic drawing from the rat brain atlas of Paxinos and Watson (69) illustrating the subdivisions of the PFC and MC at the bregma of 3.72 mm. **(B)** Low-magnification photomicrographs capturing representative coronal sections through the PFC and MC of 5-week-old spontaneously hypertensive rats (SHRs). Key regions include the prelimbic (PRL), cingulate (CG1), lateral orbitofrontal (LO), ventral orbitofrontal (VO), primary motor (M1), and secondary motor (M2) cortices. *Scale bar*, 1 mm.

**Figure 2:**
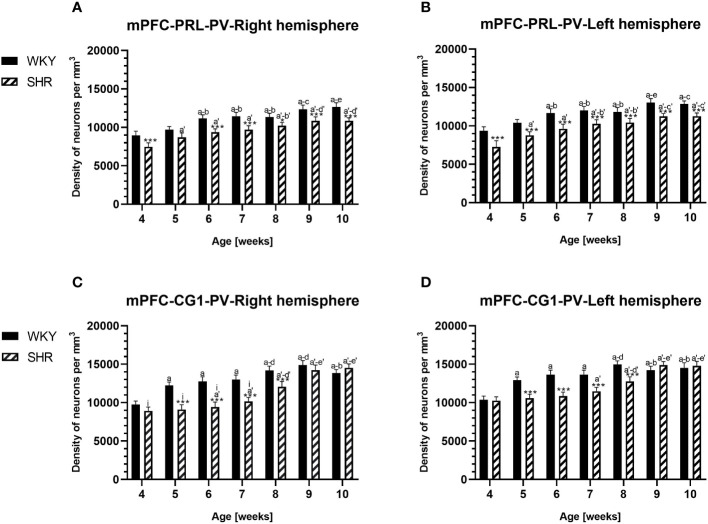
Densities of parvalbumin (PV)-expressing neurons in the prelimbic (PRL) cortex **(A, B)** and the cingulate (CG1) cortex **(C, D)** in Wistar Kyoto rats (WKYs) and spontaneously hypertensive rats (SHRs) during postnatal development. Data are expressed as the mean ± SD (n = 5 or 6). *p ≤ 0.05; ***p ≤ 0.001 (statistically significant differences between WKYs and SHRs). Age-dependent differences show the following: *a–f-* developmental differences (*p* < 0.05–*p* < 0.001) in the WKY strain; *a'–f'-* developmental differences (*p* < 0.05–*p* < 0.001) in the SHR strain; *a, a'-* 4 weeks vs. subsequent weeks; *b, b'-* 5 weeks vs. subsequent weeks; *c, c'-* 6 weeks vs. subsequent weeks; *d, d'-* 7 weeks vs. subsequent weeks; *e, e'-* 8 weeks vs. subsequent weeks; *f, f'-* 9 weeks vs. 10 weeks; and *i-* differences between the right and left hemispheres.

**Figure 3:**
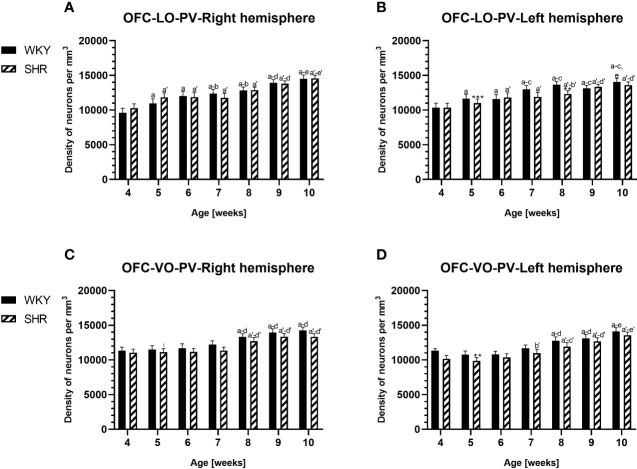
Densities of parvalbumin (PV)-expressing neurons in the lateral orbitofrontal (LO) cortex **(A, B)** and ventral orbitofrontal (VO) cortex **(C, D)** in Wistar Kyoto rats (WKYs) and spontaneously hypertensive rats (SHRs) during postnatal development. Data are expressed as the mean ± SD (n = 5 or 6). **p ≤ 0.01; ***p ≤ 0.001 (statistically significant differences between WKYs and SHRs). Age-dependent differences show the following: *a–f-* developmental differences (*p* < 0.05–*p* < 0.001) in the WKY strain; *a'–f'-* developmental differences (*p* < 0.05–*p* < 0.001) in the SHR strain; *a, a'-* 4 weeks vs. subsequent weeks; *b, b'-* 5 weeks vs. subsequent weeks; *c, c'-* 6 weeks vs. subsequent weeks; *d, d'-* 7 weeks vs. subsequent weeks; *e, e'-* 8 weeks vs. subsequent weeks; *f, f'-* 9 weeks vs. 10 weeks; and *i-* differences between the right and left hemispheres.

**Figure 4:**
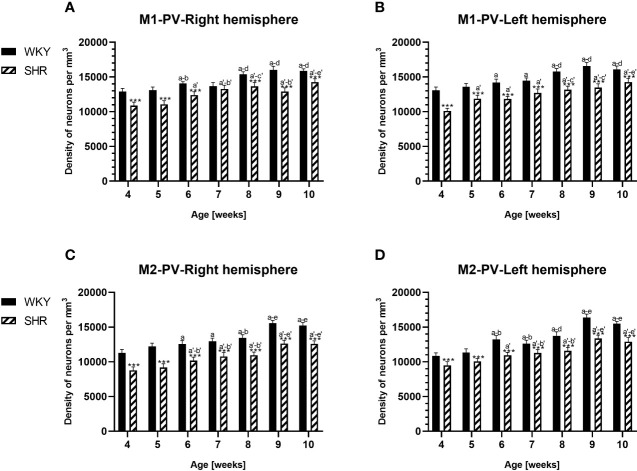
Densities of parvalbumin (PV)-expressing neurons in the primary motor (M1) cortex **(A, B)** and secondary motor (M2) cortex **(C, D)** in Wistar Kyoto rats (WKYs) and spontaneously hypertensive rats (SHRs) during postnatal development. Data are expressed as the mean ± SD (n = 5 or 6). ***p ≤ 0.001 (statistically significant differences between WKYs and SHRs). Age-dependent differences show the following: *a–f-* developmental differences (*p* < 0.05–*p* < 0.001) in the WKY strain; *a'–f'*- developmental differences (*p* < 0.05–*p* < 0.001) in the SHR strain; *a*, *a*'- 4 weeks vs. subsequent weeks; *b*, *b'-* 5 weeks vs. subsequent weeks; *c*, *c'-* 6 weeks vs. subsequent weeks; *d, d'-* 7 weeks vs. subsequent weeks; *e, e'-* 8 weeks vs. subsequent weeks; and *f, f'-* 9 weeks vs. 10 weeks.

**Figure 5:**
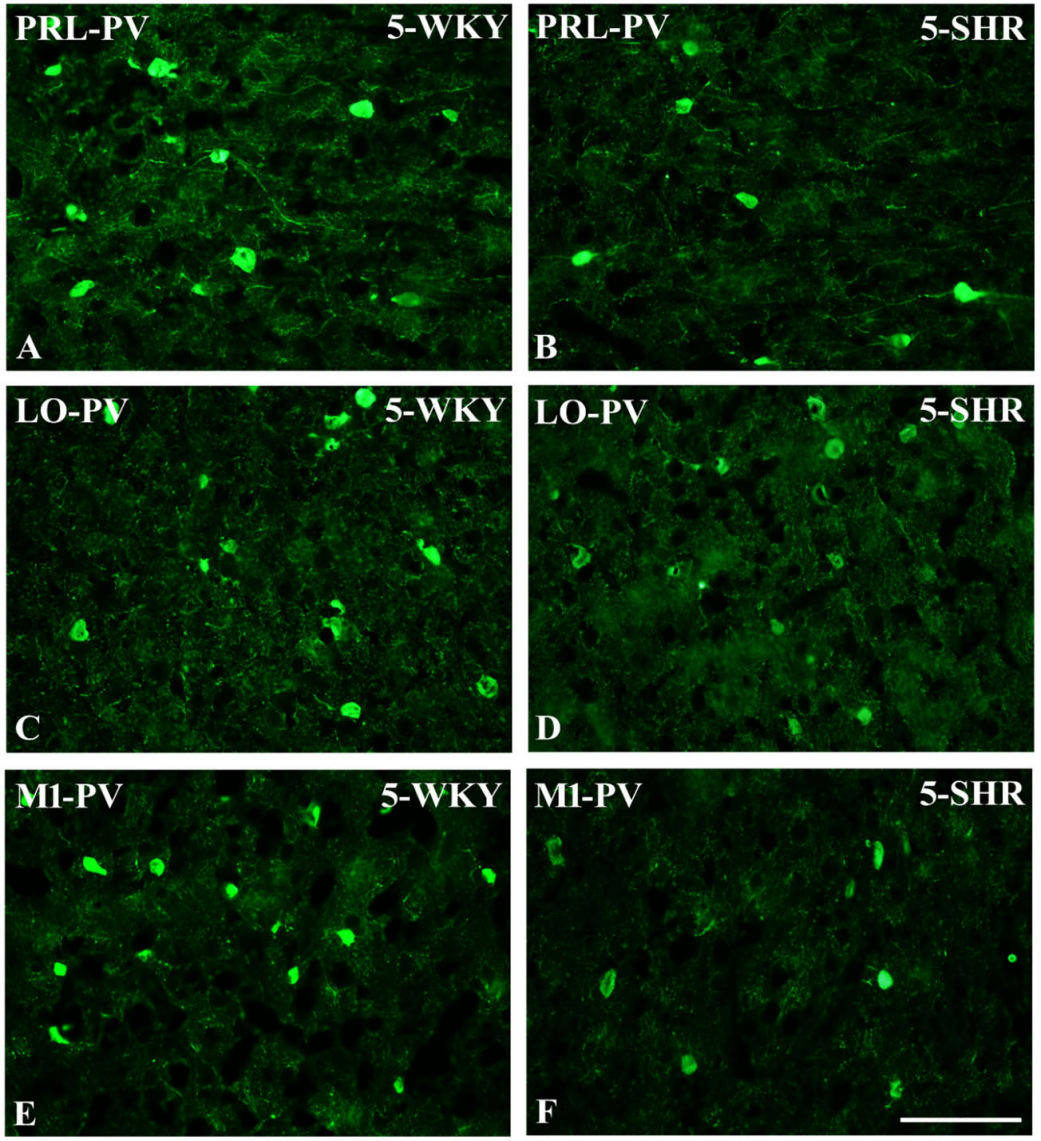
Representative color photomicrographs depicting the staining patterns of parvalbumin (PV)-expressing neurons in the prelimbic (PRL), lateral orbitofrontal (LO), and primary motor (M1) cortices of Wistar Kyoto rats (WKYs) **(A, C, E)** and spontaneously hypertensive rats (SHRs) **(B**, **D, F)**. Significantly diminished cell densities were observed in the PRL and M1 of 5-week-old SHRs **(B, F)** in comparison to age-matched WKYs **(A, E)**. Notably, no significant differences were observed in the LO cortex of 5-week-old WKYs **(C)** and SHRs **(D)**. *Scale bar*, 200 μm.

**Figure 6:**
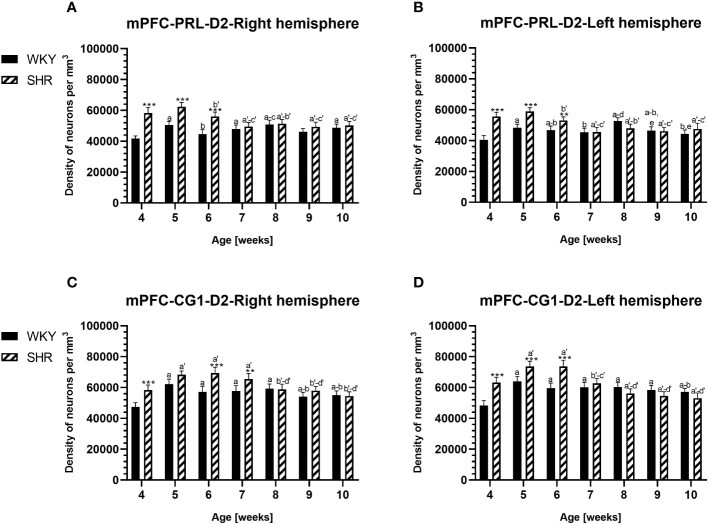
Densities of neurons enriched in dopamine receptor subtype 2 (D2) in the prelimbic (PRL) cortex **(A, B)** and cingulate (CG1) cortex **(C, D)** in Wistar Kyoto rats (WKYs) and spontaneously hypertensive rats (SHRs) during postnatal development. Data are expressed as the mean ± SD (n = 5 or 6). **p ≤ 0.01, ***p ≤ 0.001 (statistically significant differences between WKYs and SHRs). Age-dependent differences show the following: *a–f-* developmental differences (*p* < 0.05–*p* < 0.001) in the WKY strain; *a'–f'-* developmental differences (*p* < 0.05–*p* < 0.001) in the SHR strain; *a, a'-* 4 weeks vs. subsequent weeks; *b, b'-* 5 weeks vs. subsequent weeks; *c, c'-* 6 weeks vs. subsequent weeks; *d, d'-* 7 weeks vs. subsequent weeks; *e, e'-* 8 weeks vs. subsequent weeks; and *f, f'-* 9 weeks vs. 10 weeks. Data for PRL and CG1 in 5- and 10- week-old WKYs and SHRs were earlier published in our study (61) and were included here only to complete the pattern of postnatal development.

**Figure 7:**
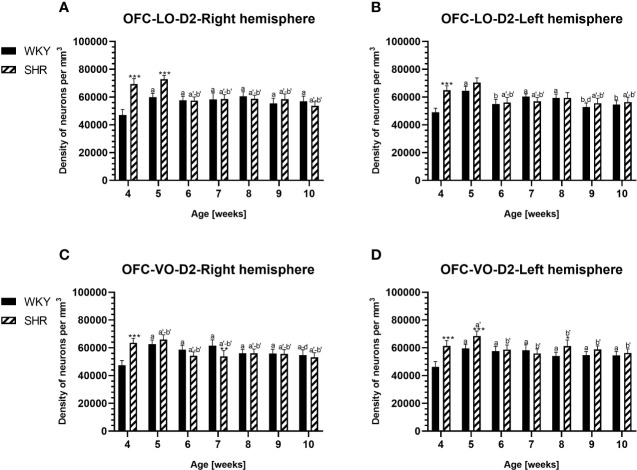
Densities of neurons enriched in dopamine receptor subtype 2 (D2) in the lateral orbitofrontal (LO) cortex **(A, B)** and ventral orbitofrontal (VO) cortex **(C, D)** in Wistar Kyoto rats (WKYs) and spontaneously hypertensive rats (SHRs) during postnatal development. Data are expressed as the mean ± SD (n = 5 or 6). *p ≤ 0.05; **p ≤ 0.01; ***p ≤ 0.001 (statistically significant differences between WKYs and SHRs). Age-dependent differences show the following: *a–f-* developmental differences (*p* < 0.05–*p* < 0.001) in the WKY strain; *a'–f'-* developmental differences (*p* < 0.05–*p* < 0.001) in the SHR strain; *a, a'-*, 4 weeks vs subsequent weeks; *b, b'-* 5 weeks vs. subsequent weeks; *c, c'-* 6 weeks vs. subsequent weeks; *d, d'-* 7 weeks vs. subsequent weeks; *e, e'-* 8 weeks. subsequent weeks; *f, f'-* 9 weeks vs. 10 weeks.

**Figure 8:**
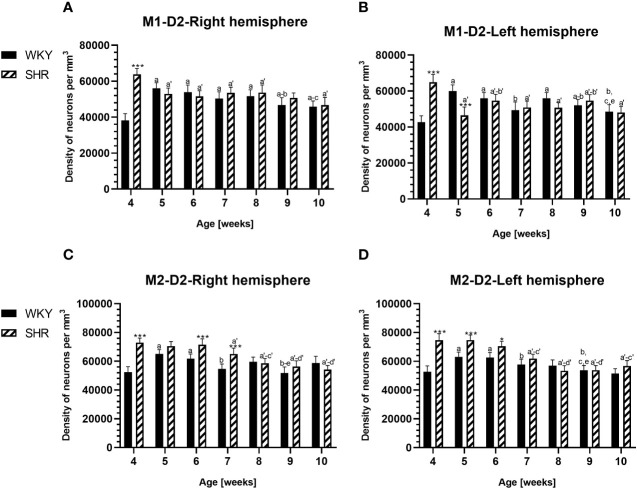
Densities of neurons enriched in dopamine receptor subtype 2 (D2) in the primary motor (M1) cortex **(A, B)** and secondary motor (M2) cortex **(C, D)** in Wistar Kyoto rats (WKYs) and spontaneously hypertensive rats (SHRs) during postnatal development. Data are expressed as the mean ± SD (n = 5 or 6). *p ≤ 0.05; ***p ≤ 0.001 (statistically significant differences between WKYs and SHRs). Age-dependent differences show the following: *a–f-* developmental differences (*p* < 0.05–*p* < 0.001) in the WKY strain; *a'–f'-* developmental differences (*p* < 0.05–*p* < 0.001) in the SHR strain; *a, a'-* 4 weeks vs. subsequent weeks; *b, b'-* 5 weeks vs. subsequent weeks; *c, c'-* 6 weeks vs. subsequent weeks; *d, d'-* 7 weeks vs. subsequent weeks; *e, e'-* 8 weeks vs. subsequent weeks; and *f, f'-* 9 weeks vs. 10 weeks.

**Figure 9:**
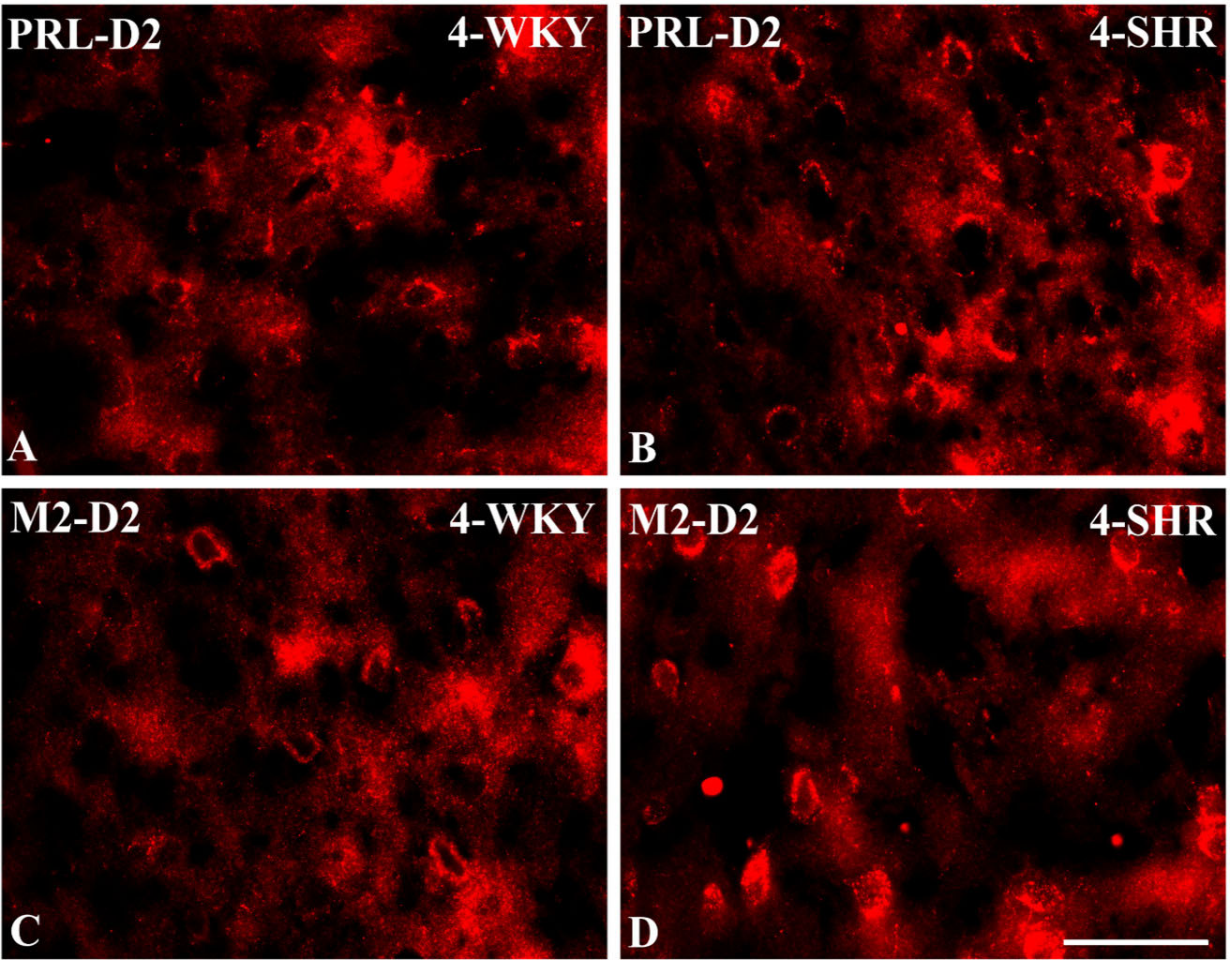
Representative color photomicrographs depicting the staining patterns of dopamine receptor subtype 2 (D2)-expressing neurons in the prelimbic (PRL) and secondary motor (M2) cortices of Wistar Kyoto rats (WKYs) **(A**, **C)** and spontaneously hypertensive rats (SHRs) **(B**, **D)**. Significantly higher densities of these cells were observed in the PRL and M2 of 4-week-old SHRs **(B, D)** in comparison to age-matched WKYs **(A, C)**. *Scale bar*, 100 µm.

**Figure 10:**
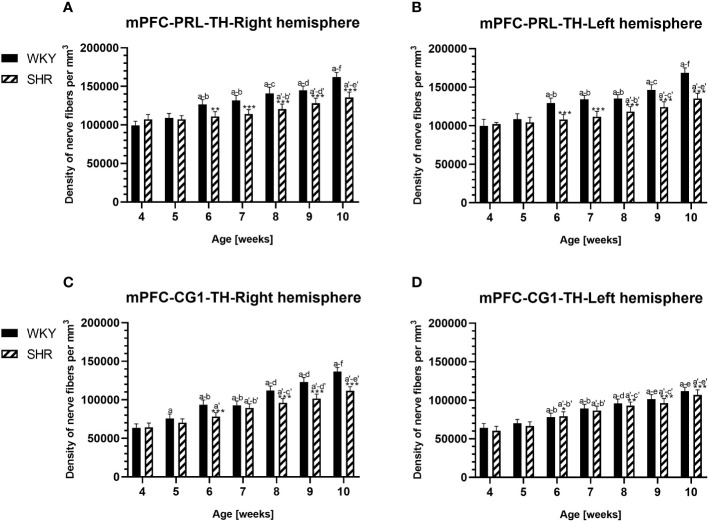
Densities of tyrosine hydroxylase (TH)-expressing fibers in the prelimbic (PRL) cortex **(A, B)** and cingulate (CG1) cortex **(C, D)** of Wistar Kyoto rats (WKYs) and spontaneously hypertensive rats (SHRs) during postnatal development. Significantly reduced fiber densities were observed in pubertal and postpubertal SHRs in comparison to age-matched WKYs. Data are expressed as the mean ± SD (n = 5 or 6). *p ≤ 0.05; **p ≤ 0.01; ***p ≤ 0.001 (statistically significant differences between WKYs and SHRs). Age-dependent differences show the following: a–f- developmental differences (*p* < 0.05–*p* < 0.001) in the WKY strain; *a'–f'-* developmental differences (*p* < 0.05–*p* < 0.001) in the SHR strain; *a, a'-* 4 weeks vs. subsequent weeks; *b, b'-* 5 weeks vs. subsequent weeks; *c, c'-* 6 weeks vs. subsequent weeks; *d, d'-* 7 weeks vs. subsequent weeks; *e, e'-* 8 weeks vs. subsequent weeks; and *f, f'-* 9 weeks vs. 10 weeks. Data for PRL and CG1 in 5- and 10-week-old WKYs and SHRs were earlier published in our study (61) and were included here only to complete the pattern of postnatal development.

**Figure 11:**
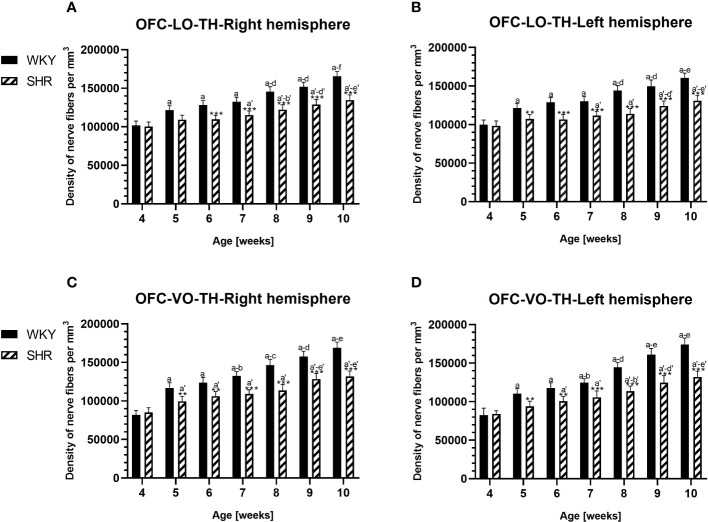
Densities of of tyrosine hydroxylase (TH)-expressing fibers in the l ateral orbitofrontal (LO) cortex **(A, B)** and ventral orbitofrontal (VO) cortex **(C, D)** in Wistar Kyoto rats (WKYs) and spontaneously hypertensive rats (SHRs) during postnatal development. Data are expressed as the mean ± SD (n = 5 or 6). **p ≤ 0.01; ***p ≤ 0.001 (statistically significant differences between WKYs and SHRs). Age-dependent differences show the following: a–f- developmental differences (*p* < 0.05–*p* < 0.001) in the WKY strain; *a'–f'-* developmental differences (*p* < 0.05–*p* < 0.001) in the SHR strain; *a, a'-* 4 weeks vs. subsequent weeks; *b, b'-* 5 weeks vs. subsequent weeks; *c, c'-* 6 weeks vs. subsequent weeks; *d, d'-* 7 weeks vs. subsequent weeks; *e, e'-* 8 weeks vs. subsequent weeks; and *f, f'-* 9 weeks vs. 10 weeks.

**Figure 12:**
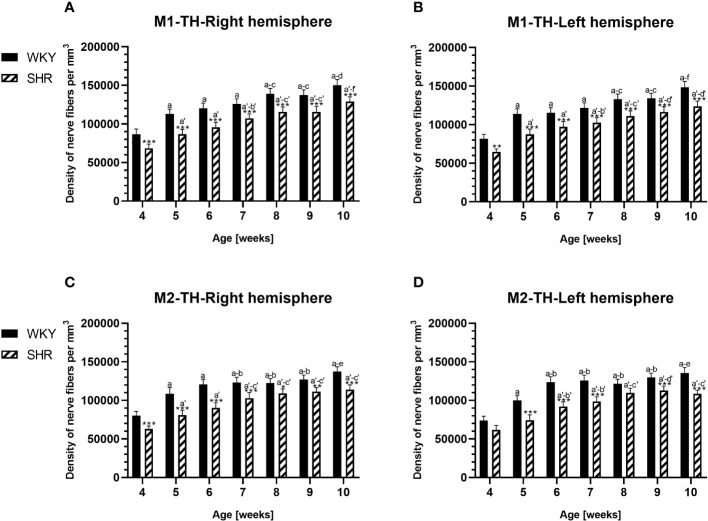
Densities of tyrosine hydroxylase (TH)-expressing fibers in the primary motor (M1) cortex **(A, B)** and secondary motor (M2) cortex **(C, D)** of Wistar Kyoto rats (WKYs) and spontaneously hypertensive rats (SHRs) during postnatal development. Data are expressed as the mean ± SD (n = 5 or 6). *p ≤ 0.05, **p ≤ 0.01; ***p ≤ 0.001 (statistically significant differences between WKYs and SHRs). Age-dependent differences show the following: *a–f-* developmental differences (*p* < 0.05–*p* < 0.001) in the WKY strain; a'–f'- developmental differences (*p* < 0.05–*p* < 0.001) in the SHR strain; *a, a'-* 4 weeks vs. subsequent weeks; *b, b'-* 5 weeks vs. subsequent weeks; *c, c'-* 6 weeks vs. subsequent weeks; *d, d'-* 7 weeks vs. subsequent weeks; *e, e'-* 8 weeks vs. subsequent weeks; *f, f'-* 9 weeks vs. 10 weeks.

**Figure 13:**
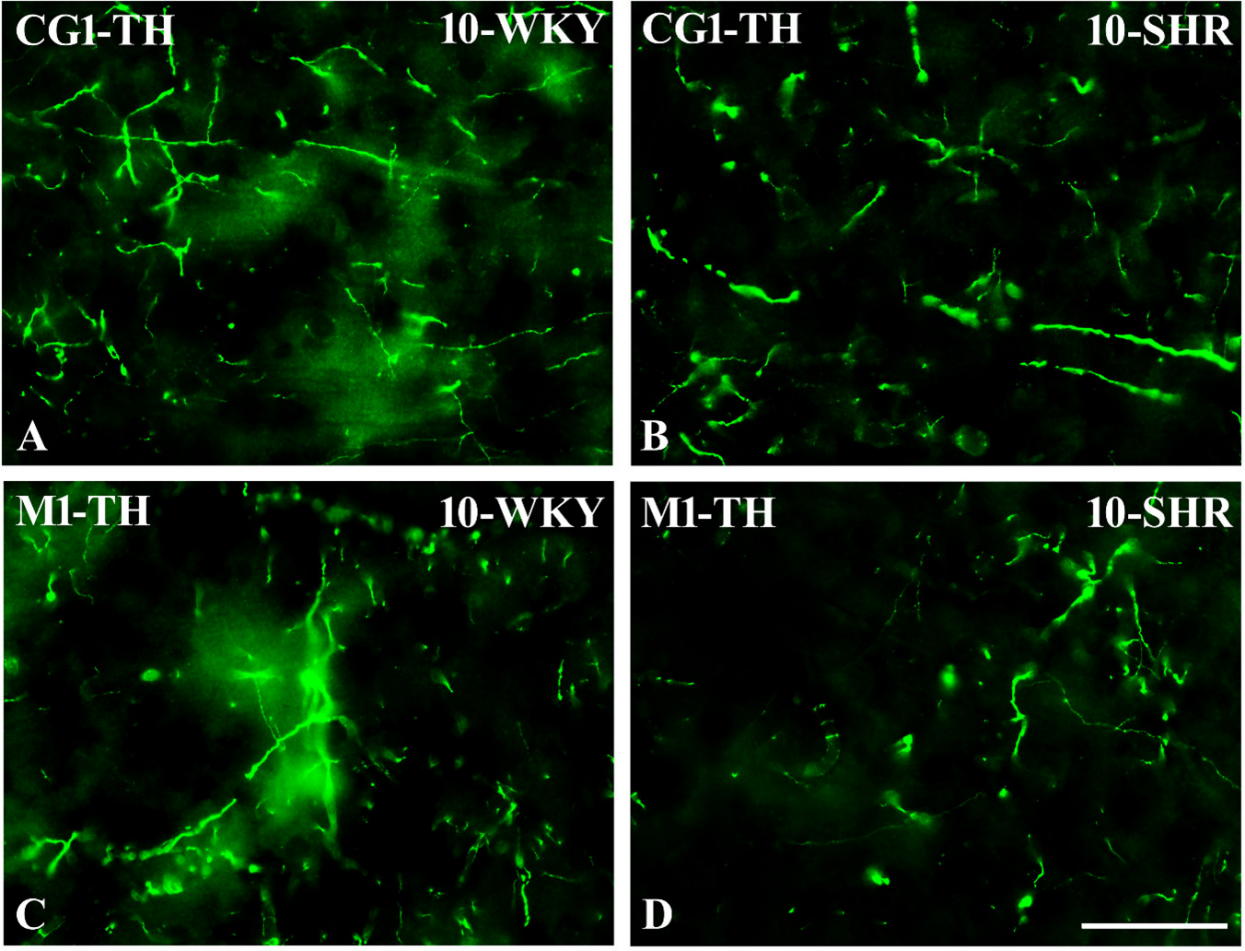
Representative color photomicrographs depicting the staining patterns of tyrosine hydroxylase (TH)-expressing fibers in the anterior cingulate (CG1) and primary motor (M1) cortices of Wistar Kyoto rats (WKYs) **(A**, **C)** and spontaneously hypertensive rats (SHRs) **(B**, **D)**. Significantly reduced fiber densities were observed in the CG1 and M1 of 10-week-old SHRs **(B, D)** as compared to age-matched WKYs (**A, C**). *Scale bar*, 100 µm.

**Figure 14:**
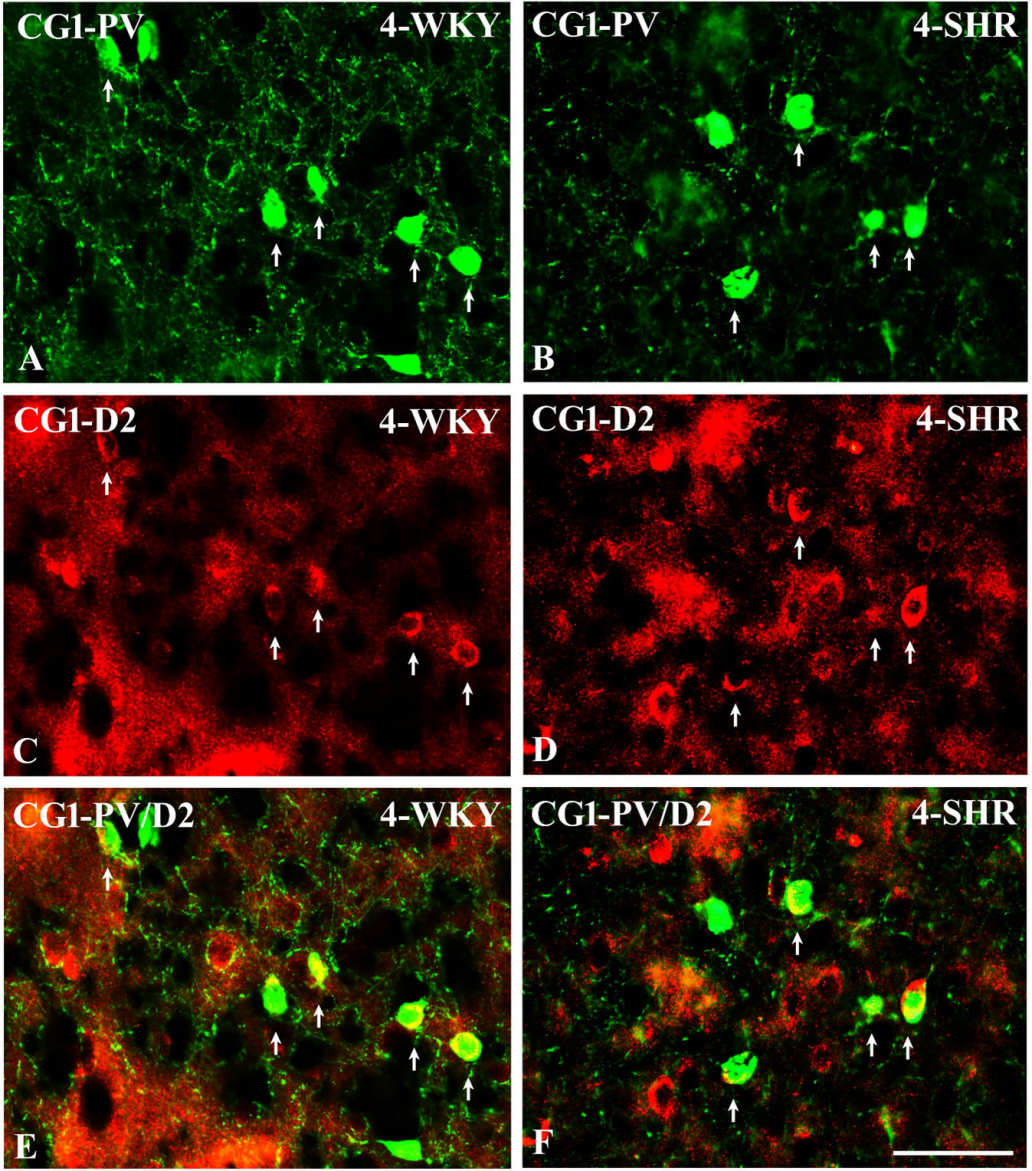
Representative color photomicrographs illustrating the anatomical relationship between parvalbumin (PV)-expressing neurons and dopamine receptor subtype 2 (D2) in the anterior cingulate (CG1) cortex of juvenile Wistar Kyoto rats (WKYs) **(A**, **C**, **E)** and spontaneously hypertensive rats (SHRs) **(B**, **D**, **F)**. Note the extensive co-localization of PV with D2 in both rat strains (*arrows*). Note also that the expression of D2 is much more abundant in SHRs **(D)** compared to age-matched WKYs **(C)**. *Scale bar*, 100 µm.

Anatomically, the rat PFC includes two main regions—the medial PFC (mPFC) with the PRL and CG1 and the ventrally located orbitofrontal PFC (oPFC) with the LO and VO—which were chosen for investigation in the present study (**Figures 1A, B**). In between these regions and dorsally lies the MC, consisting of M1 and M2, which were taken into consideration (**Figures 1A, B**). In all of these regions, the densities of neurons immunoreactive to PV and/or D2 and fibers expressing TH were evaluated and compared in WKYs and SHRs (**Figures 2**–**13**). In addition, as part of preliminary research in these regions, the relationship between PV and D2 was also elucidated (**Figure 14**).

### 3.1 Parvalbumin

The results showed that, in the PRL (**Figures 2A, B**; **5A, B**, green photomicrographs), M1 (**Figures 4A, B**; **5E, F**, green photomicrographs), and M2 (**Figures 4C, D**), the densities of PV+ cells were significantly decreased (*p* < 0.001) in SHRs when compared to WKYs at almost any age studied. The CG1 density values were also decreased (*p* < 0.001) in the SHRs, but only from 5 to 8 weeks of their lives (**Figures 2C, D**). No density changes among PV+ cells were observed in the LO and VO (*p* > 0.05), with the exception of the left hemisphere in 5-week-old SHRs where the density of PV+ neurons decreased (*p* < 0.01–0.001) in relation to WKYs (**Figures 3A–D**; **5C, D**, green photomicrographs). Moreover, the comparison of the right and left hemispheres in SHRs revealed disparities in the density of PV+ neurons. Notably, in the CG1 during weeks 4–7 (*p* < 0.01; **Figures 2C, D**), the density of neurons positive for PV in the right hemisphere was lower than that in the left hemisphere. In turn, a higher density of PV+ neurons within the right hemisphere in VO in week 5 was found (*p* < 0.01; **Figures 3C, D**).

### 3.2 Dopamine receptor 2

PV deficits were accompanied by D2 upregulation, which were observed mostly in prepubertal SHRs. In the PRL (**Figures 6A, B**; **9A, B**, red photomicrographs), CG1 (**Figures 6C, D**), and M2 (**Figures 8C, D**; **9C, D**, red photomicrographs), the densities of D2+ cells were significantly elevated (*p* < 0.01–0.001) in 4-, 5-, and/or 6-week-old SHRs in comparison to age-matched WKYs; however, in older animals, the densities were similar (*p* > 0.05) in both rat strains. In the case of LO (**Figures 7A, B**) and VO (**Figures 7C, D**), the density values were also increased (*p* < 0.001), but only in 4-, 5- and/or 6-week-old SHRs. Significantly elevated D2+ density values (*p* < 0.001) were observed in M1 only in 4-week-old SHRs (**Figures 8A, B**).

### 3.3 Hydroxylase tyrosine

In all studied regions, PV deficits were also accompanied by the downregulation of TH, which was observed mostly in pubertal and postpubertal SHRs. In M1 (**Figures 12A, B**; **13C, D**, green photomicrographs) and M2 (**Figures 12C, D**), the densities of TH+ fibers were significantly decreased (*p* < 0.01 and 0.001) in SHRs in comparison to WKYs at any age studied. In VO (**Figures 11C, D**), decreased (*p* < 0.05 and 0.001) density values were observed in 5- to 10-week-old SHRs; however, in 4-week-old animals, the values were comparable (*p* > 0.05) in both rat strains. In the PRL (**Figures 10A, B**), CG1 (**Figures 10C, D**, **13A, B**, green photomicrographs), and LO (**Figures 11A, B**), the density values were decreased in 6- to 10-week-old SHRs only (*p* < 0.01).

### 3.4 PV/D2 relationships

To elucidate the relationship between PV+ cells and D2+ elements, some sections in each subject were processed for double immunofluorescence staining. The results revealed extensive co-expression of D2 in PV+ neurons in both WKYs and SHRs at any age studied. The co-expression patterns of PV and D2 in various regions of the PFC and MC did not differ significantly between WKYs and SHRs and ranged from 60% in PRL to ~75% in M1. However, a higher abundance of these proteins on PV+ cells in SHRs was easily recognizable (**Figures 14A–F**).

### 3.5 Summary of results

Our findings, which exhibited a region-specific reduction in PV+ cell density along with D2 receptor upregulation and TH downregulation in SHRs, serve as a crucial starting point for more comprehensive research aimed at unraveling the pathophysiology of ADHD. These baseline studies have identified specific neurobiological alterations within the PFC and MC regions, shedding light on the intricate interplay between the inhibitory and excitatory neurotransmission systems. The reduction in the density of PV+ cells implies a potential imbalance in inhibitory signaling, while the observed upregulation of D2 and downregulation of TH suggest alterations in the dopaminergic system, which is crucial for attention, impulse control, and motor function. Building upon these foundational findings, further detailed research can delve into the molecular, cellular, and circuit-level mechanisms underlying these alterations. Investigating the specific interactions between PV+ interneurons, D2 receptors, and the DA synthesis pathway in ADHD-relevant brain regions could provide a more nuanced understanding of the neurobiological basis of this disorder.

## 4 Discussion

The results presented here provide evidence that, in the PFC and MC, the densities of PV+ interneurons, which in the cerebral cortex are the main subset of inhibitory GABAergic neurons (70), were significantly reduced in pre- and postpubertal SHRs, a validated ADHD animal model (40). This parallels the diminished GABA content in pediatric ADHD patients and aligns with the reduced SICI observed in ADHD. Notably, PV+ deficits in all the studied regions coincided with D2 receptor upregulation, primarily in prepubertal SHRs, and TH downregulation, predominantly in pubertal and postpubertal SHRs. Given that PV+ neurons co-expressed substantial D2 receptors (present results) (29, 33), their upregulation could exacerbate the deficits in GABA and impairments in cortical inhibition. In addition, downregulation of TH, a key enzyme in DA synthesis, could contribute to reduced GABA neurotransmission and behavioral inhibition deficits. The intricate interplay between GABAergic transmission and DA signaling in the PFC and MC underscores their pivotal role in sensory information filtering and behavioral response determination (71). A comprehensive understanding of these mechanisms during juvenile SHRs development offers critical insights into the neurobiological foundations of ADHD, with implications for targeted interventions.

The findings obtained unequivocally illustrate substantial PV deficits in SHRs across diverse regions of the PFC and MC, except for the LO and VO regions. This observation resonates with previous research highlighting diminished GABA concentrations in the PFC and MC of children diagnosed with ADHD compared to their typically developing counterparts (22, 24). The identified PV deficits align with documented impairments in SICI in individuals with ADHD, suggesting a GABAergic deficiency within this population (27). Given that SICI modulation involves GABA-A agonists and is believed to be orchestrated by GABA-A cortical interneurons (72), it appears that fast-spiking PV+ neurons are the most plausible candidates to meet both criteria (28, 70). These neurons exhibit GABAergic properties and boast abundant GABA-A receptors (29), making them prime contenders for playing a crucial role in the observed deficits. Recent evidence has emphasized the pivotal role of PV+ interneurons in regulating pyramidal neuron activity (73, 74), modulating the excitation/inhibition (E/I) balance (75, 76) to drive appropriate behavioral responses (77, 78). Consequently, deficits in PV+ cell density could disrupt the E/I balance, potentially increasing excitatory drive and contributing to inappropriate behaviors or psychiatric disorders (79). Good examples of such phenomena are schizophrenia and autism spectrum disorder, which is characterized by significant deficits in GABAergic signaling and reduced expressions of GAD-67 and/or PV (80, 81). It also appears that at least some of the ADHD symptoms may have a similar etiology. An example is the correlation between GABA concentrations and motor control in healthy adults (82). ADHD patients and SHRs showed increased motor activity, with reduced GABA in the MC of human patients (22) and PV+ deficits in the MC of SHRs (present results). There is also evidence that a low GABA concentration in the CG1 is strongly associated with high inattention scores in children with ADHD (24). Interestingly, SHRs that also displayed inattention had PV+ deficits in the CG1 (present results). Notably, the severity of ADHD symptoms and the motor skill proficiency of school-aged children with this disorder have been linked to reduced SICI in the MC, indicating a GABA-A/PV deficit (27). Recent reports have also suggested that the glutamate levels in the PFC may be related to the intensity of ADHD traits in human patients and in SHRs (83, 84). Elevated glutamate levels in the CG1 of patients with ADHD positively correlate with the severity of symptoms related to hyperactivity and impulsivity (84).

The current study reveals a noteworthy association between deficits in PV+ and the increased density of neurons expressing D2 receptors in various regions of the PFC and MC in SHRs, particularly evident in prepubertal individuals. While some prior investigations indicated no significant differences in the expression of D1 and D2 receptors between SHRs and WKYs (85–87), multiple studies have consistently reported the upregulation of D1 and D2 receptors in various brain regions of SHRs, including the frontal cortex, nucleus accumbens, and striatum (88–91). These receptors in the cortical areas are localized both in the pyramidal glutamatergic neurons and in various classes of GABAergic interneurons (present results) (29, 92). Considering that, in physiological conditions, a high proportion of PV+ interneurons co-express both D1 or D2 receptors (33, 93) and DA primarily increases PV+ cell excitability, enhancing the GABAergic transmission *via* D1 activation to suppress persistent firing of pyramidal neurons (35), increased D2 expression on these cells in juvenile SHRs may favor opposite consequences. Thus, the overexpression of D2 in conjunction with the overall deficit of PV+ expression in the PFC and MC of SHRs could, at least in part, lead to the reduced behavioral inhibition observed in ADHD-affected individuals. This aligns with the established roles of D2 receptors in mediating hyperactivity and responses to amphetamine, phenomena observed in both ADHD-affected individuals and animal models (94). Notably, targeted deletion of the D2 (but not the D3 or D4) DA receptor in coloboma mice, an ADHD mouse model, eliminated hyperactivity, and similar effects were observed in response to amphetamine treatment in both coloboma mice and human ADHD patients (94–97). The observed upregulation of D2 receptors in the 4- to 6-week-old SHRs in our study aligns temporally with the manifestation of ADHD symptoms in both rats and human patients. Rats undergo weaning at approximately 3 weeks, with puberty commencing around 7–8 weeks. In comparison, humans are typically weaned around 6 months, with puberty beginning at approximately 11.5 years. The 4- to 6-week-old SHRs in our study, equivalent to 7–10 years of age in children (98), exhibited an upregulation of D2 receptors in the PFC and MC. The age of 7 weeks in rats, when the contents of D2 receptors for the two strains became comparable, corresponds to the onset of puberty. This temporal alignment closely mirrors clinical findings in children with ADHD, where symptoms manifest in young school-aged individuals and hyperactivity tends to improve after puberty (2).

Preliminary data indicating the co-localization of PV and D2 in PFC and MC neurons further add to the complexity of the findings. While detailed analysis on this topic is lacking in the literature, future investigations exploring the interaction between D2 receptors and PV+ neurons in both WKYs and SHRs may provide valuable insights into the neurobiological mechanisms at play in ADHD.

The current findings reveal PV deficits in the PFC and MC regions of SHRs, accompanied by a reduction in TH+ fibers, predominantly observed in pubertal and postpubertal SHRs. Previous reports documented decreased TH protein and mRNA levels in the PFC of SHRs (39, 99–101), but data on this aspect in human ADHD patients are lacking. As the gene encoding TH appears to be not altered in individuals with ADHD, the phenomenon of TH downregulation is difficult to explain. There is also no information on the content of l-3,4-dihydroxyphenylalanine (l-DOPA; a precursor of DA synthesis) in human ADHD patients and in SHRs that could shed light on the role of TH downregulation in the pathogenesis of ADHD. However, TH deficits may have an impact on the overall DA action in the PFC and MC as methylphenidate treatment increased the TH levels in SHRs and improved the ADHD symptoms in these animals (100). It is worth mentioning that, in the case of PFC, one factor should be considered when comparing the TH levels in SHRs and WKYs, or in human ADHD patients. The PFC undergoes a prolonged maturation process, exhibiting structural and connectivity changes that extend through adolescence into early adulthood (102–104). This protracted development of the DA input to the PFC is attributed to the continued growth of DA axons during adolescence (105). Notably, both ADHD human patients and SHRs display a maturational trajectory of the PFC that is typical but delayed by a few years or weeks, respectively (106, 107). This delay is also evident in the maturation of dopaminergic pathways. When comparing the TH levels in the PFC between SHRs and WKYs, it is essential to consider developmental delay. The presence of age-inappropriate levels of hyperactivity/impulsivity and inattention in both ADHD individuals and SHRs may reflect this delayed maturation of the PFC in these populations (107).

The study’s focus on the temporal alignment of neurotransmitter alterations with ADHD symptom manifestation provides crucial insights into the developmental trajectory of this disorder. While acknowledging the study’s merit in shedding light on the neurobiological foundations of ADHD, several limitations exist. The existence of a PFC in rats and its primate equivalent has been a subject of ongoing debate. Anatomical studies suggest that the rat medial PFC is homologic to both the primate anterior cingulate cortex (ACC) and the dorsolateral PFC (108, 109). Functionally, in rats, it has been found that there are strong correlations between motor planning, movement, and reward anticipation, similar to observations in the primate ACC (110). Moreover, it is well known that rats may encode information over delays by utilizing body posture or variations in the running path that are tracked by medial PFC neurons (111, 112). Based on these results, the rat medial PFC appears to combine elements of the primate ACC and dorsolateral PFC (111–113). To aim for a deeper knowledge of the homology of PFC across species, further research is required to gather more data. In addition, the study calls for continued exploration of the interactions of PV+ neurons with D2 receptors in WKYs and SHRs, emphasizing the need for more in-depth cross-species homology investigations. In conclusion, this research marks a significant stride toward unraveling the complexities of ADHD neurobiology, emphasizing the importance of PV+ interneurons, D2 receptors, and TH in shaping the neurotransmission dynamics. The findings of this study not only contribute to existing knowledge but also underscore the need for more extensive research to unveil etiological factors, clinical ramifications, and potential therapeutic interventions for ADHD. These outcomes hold promise for advancing targeted treatments and facilitating more effective management of this prevalent neurodevelopmental disorder.

## 5 Conclusions

The results of the present study provide evidence that, in the PFC and MC, the density of PV+ neurons, which form in the cerebral cortex as the main subset of inhibitory GABAergic neurons, is significantly reduced in pre- and postpubertal SHRs. Thus, these results unequivocally align with prior findings that have consistently reported significantly reduced GABA content in children with ADHD (22, 114). Moreover, they firmly corroborate the observed pattern of diminished SICI in ADHD (27). In addition, in all studied areas, the PV+ deficits were accompanied by the upregulation of D2 observed mostly in prepubertal SHRs and the downregulation of TH observed mostly in pubertal and postpubertal SHRs. As cortical PV+ neurons co-express large amounts of D2 receptors (present results), which selectively suppress GABAergic transmission (115), the upregulation of these proteins could additionally potentiate deficits in GABA and/or deficits in cortical inhibition. Similarly, indirect effects on GABA neurotransmission and behavioral inhibition may also have downregulation of TH, which is considered to be the rate-limiting enzyme of DA synthesis. Nevertheless, additional research is imperative to comprehensively substantiate the precise etiological factors, repercussions, and broader implications of impaired GABAergic signaling in the pathophysiology of individuals with ADHD. Robust investigations are necessary to deepen our understanding for more effective treatment of this disorder.

## Data availability statement

The raw data supporting the conclusions of this article will be made available by the authors, without undue reservation.

## Ethics statement

The animal study was approved by the Local Ethical Commission of the University of Warmia and Mazury in Olsztyn (no. 43/2014). The study was conducted in accordance with the local legislation and institutional requirements.

## Author contributions

EBC: Data curation, Formal analysis, Investigation, Visualization, Writing – original draft. AK: Conceptualization, Data curation, Formal analysis, Funding acquisition, Investigation, Methodology, Resources, Supervision, Writing – review & editing. MR: Conceptualization, Data curation, Formal analysis, Investigation, Methodology, Validation, Writing – original draft. ACHW: Writing – review & editing.
